# Supplementary material for: Novel Driver Strength Index highlights important cancer genes in TCGA PanCanAtlas patients
Source: PeerJ. 2022 Aug 11;10:e13860. doi: 10.7717/peerj.13860 (PMC9375969; doi:10.7717/peerj.13860)
Supplement: Supplemental Information 2 [file peerj-10-13860-s002.zip › COHORTS/genes plots/detailed/2021_8_16_14_9_distribution_events_detailed_females_2.pdf]

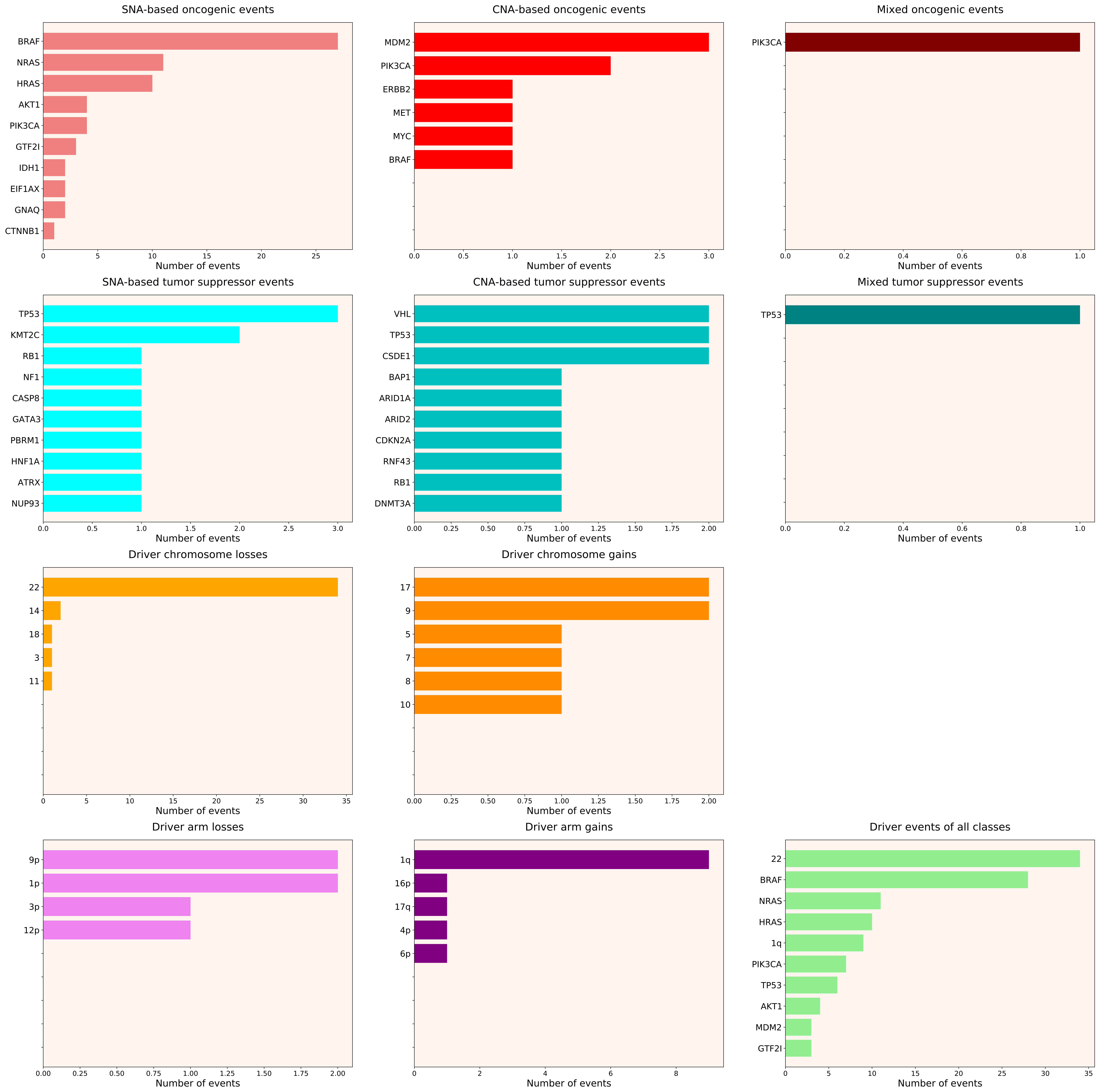

SNA-based oncogenic events
BRAF
NRAS
HRAS
AKT1
PIK3CA
GTF2I
IDH1
EIF1AX
GNAQ
CTNNB1
0 5 10 15 20 25
Number of events
CNA-based oncogenic events
MDM2
PIK3CA
ERBB2
MET
MYC
BRAF
0.0 0.5 1.0 1.5 2.0 2.5 3.0
Number of events
Mixed oncogenic events
PIK3CA
0.0 0.2 0.4 0.6 0.8 1.0
Number of events
SNA-based tumor suppressor events
TP53
KMT2C
RB1
NF1
CASP8
GATA3
PBRM1
HNF1A
ATRX
NUP93
0.0 0.5 1.0 1.5 2.0 2.5 3.0
Number of events
CNA-based tumor suppressor events
VHL
TP53
CSDE1
BAP1
ARID1A
ARID2
CDKN2A
RNF43
RB1
DNMT3A
0.00 0.25 0.50 0.75 1.00 1.25 1.50 1.75 2.00
Number of events
Mixed tumor suppressor events
TP53
0.0 0.2 0.4 0.6 0.8 1.0
Number of events
Driver chromosome losses
22
14
18
3
11
0 5 10 15 20 25 30 35
Number of events
Driver chromosome gains
17
9
5
7
8
10
0.00 0.25 0.50 0.75 1.00 1.25 1.50 1.75 2.00
Number of events
Driver arm losses
9p
1p
3p
12p
0.00 0.25 0.50 0.75 1.00 1.25 1.50 1.75 2.00
Number of events
Driver arm gains
1q
16p
17q
4p
6p
0 2 4 6 8
Number of events
Driver events of all classes
22
BRAF
NRAS
HRAS
1q
PIK3CA
TP53
AKT1
MDM2
GTF2I
0 5 10 15 20 25 30 35
Number of events
